# Environmental Exposure and Pediatric Asthma Prevalence in Saudi Arabia: A Cross-Sectional Study

**DOI:** 10.7759/cureus.46707

**Published:** 2023-10-09

**Authors:** Abdulsalam Aleid, Rayan A Alolayani, Raghad Alkharouby, Areej R Al Gawez, Faisal D Alshehri, Renad A Alrasan, Raghad Salman Alsubhi, Abbas Al Mutair

**Affiliations:** 1 Neurosurgery, King Faisal University, Al Ahsa, SAU; 2 College of Medicine, Taif University, Taif, SAU; 3 Collage of Medicine, King Saud Bin Abdulaziz University for Health Sciences, Jeddah, SAU; 4 Pediatric Medicine, Vision Colleges, Qatif, SAU; 5 College of Medicine, King Khalid University, Abha, SAU; 6 College of Medicine, Imam Abdulrahman Bin Faisal University, Dammam, SAU; 7 College of Medicine, Umm Al-Qura University, Makkah, SAU; 8 Research Center, Almoosa Specialist Hospital, Al Ahsa, SAU

**Keywords:** saudi arabia, risk factors, environmental exposure, pediatrics, asthma

## Abstract

Introduction: Asthma is a chronic respiratory disease characterized by recurrent episodes of wheezing and shortness of breath. Currently, there is no cure for asthma. However, through accurate diagnosis, appropriate therapy, and effective management strategies, individuals with asthma can achieve a better quality of life. This study focused on determining the prevalence and environmental risk factors of pediatric asthma among children in Saudi Arabia.

Methods: This cross-sectional study was undertaken from January 2023 to August 2023, encompassing five key regions in Saudi Arabia: Central, Eastern, Northern, Southern, and Western. A structured online questionnaire was disseminated to parents/guardians of children aged 2-18 with a confirmed diagnosis of asthma. The objective was to garner insights regarding pediatric asthma prevalence and associated risk factors within these regions. The questionnaire was designed with considerations for sociodemographic factors, environmental exposures, and known asthma triggers. From the survey's distribution, a total of 1666 responses were accumulated and processed for further analysis.

Results: The survey saw the participation of 1666 respondents. The age bracket of 8-13 years had the highest representation, accounting for 52.5% of the total participants. From the total cohort, 26.9% had been diagnosed with pediatric asthma. It was also observed that 56.7% of the participants resided in areas marked by high traffic or were proximate to busy roads. Additionally, 30.7% of the respondents pinpointed specific times in the year when asthma symptoms intensified. When dissecting the data, it was discerned that there were statistically significant associations between the prevalence of pediatric asthma, gender, and the regions of Saudi Arabia with reference to air pollution exposure. Notably, gender had an odds ratio of 1.12, while the regional distribution held an odds ratio of 1.

Conclusion: Our study vividly highlights the intricate landscape of pediatric asthma across Saudi Arabia, indicating its prevalence and drawing attention to associated risk factors. Noteworthy observations include the pronounced impact of gender and regional variances, particularly concerning air pollution exposure, on asthma incidence. The insights gleaned from this research are invaluable in steering the formulation and implementation of strategic interventions geared towards bolstering children's health and enhancing their life quality in regions bearing the brunt of pediatric asthma.

## Introduction

Bronchial asthma (BA) stands as the most common chronic illness in the pediatric age group, wielding profound implications on public health, encompassing both morbidity and mortality [1]. In children diagnosed with BA, the inflammation of the airways makes them hypersensitive and readily reactive to allergens and irritants. Such a heightened reactivity often manifests in distinct symptoms, notably wheezing and breathlessness [2]. While asthma remains an ailment without a definitive cure, its impacts can be significantly mitigated with timely diagnosis, adherence to prescribed medication, consistent follow-ups, and a robust management plan focusing on trigger avoidance [3].

In the global panorama, there's a discernible surge in the prevalence of BA, especially among children [4]. Within Saudi Arabia's context, although several epidemiological studies have been directed at delineating the prevalence of BA in children, results have shown variability, with reported prevalences fluctuating between 8-25% in the last thirty years [5]. This escalating trend is believed to resonate with shifts in lifestyles and augmented environmental exposures [6]. Attributes like socioeconomic dynamics, urbanization, changing dietary patterns, increasing encounters with air pollutants from sources like vehicular emissions and industrial outputs, exposure to tobacco smoke, the influx of sandstorms and dust, and heightened interaction with domestic pets, among other allergens and contaminants, have emerged as pivotal in influencing both the onset and exacerbation of BA [7-10].

Our study endeavors to explore the connection between certain environmental exposures and BA prevalence among children in Saudi Arabia. Specifically, it delves into the increasingly popular trend of owning domestic pets and its potential influence on BA prevalence, steering away from globally acknowledged correlations, thereby presenting a fresh perspective on the subject.

## Materials and methods

Study area and design

The study spanned across the entirety of Saudi Arabia, encapsulating five distinct regions: Central, Eastern, Northern, Southern, and Western. This research was anchored in a cross-sectional design and extended from August 2022 to August 2023. The data collation mechanisms integrated both online and in-person questionnaires directly from the child or their guardian.

Study setting

The research was orchestrated under the aegis of King Faisal University, drawing on their resources and extensive networks to ensure a comprehensive reach across various regions of Saudi Arabia. This ensured the inclusion of participants from diverse settings such as emergency rooms, clinics, and the general public.

Study population and sampling

The study zeroed in on children aged 2-14 years diagnosed with asthma. Given that the pediatric age in Saudi Arabia culminates at 14 years, our focus was adjusted accordingly. The total sample size was 1666. Arriving at this figure factored in a 95% confidence interval, a margin of error of ±5%, and an expected asthma rate of 385. Convenience sampling was the method of choice, cherry-picking participants based on their accessibility and willingness. The age cap was set at 14 years in alignment with the regional pediatric definition, and the decision to exclude children below 2 years was informed by the potential challenges in diagnosing asthma reliably in this age group. Those not meeting the criteria, unwilling to partake, or unable to give informed consent were excluded.

Data collection

The International Study of Asthma and Allergies in Childhood (ISAAC) questionnaire, tailored to the study's requirements, was the cornerstone for data collection. This questionnaire was tri-segmented, delving into demographics, outdoor air pollution exposure, and indoor allergen interaction. Strict measures ensured data access was exclusive to the research team, upholding the sanctity of participant confidentiality.

Study variables

The independent variables encapsulated demographics such as age, gender, educational background, professional status, and region. Conversely, the dependent variables zoomed in on asthma indicators and respiratory safeguards, underscoring exposures to external air pollutants (like traffic and industrial sites) and indoor allergens (like flora and dust mites).

Preliminary assessment

A precursor pilot study involving 20 participants was rolled out before the primary data-gathering phase. This step was instrumental in affirming the questionnaire's dependability and lucidity, leading to subtle content refinements.

Ethical considerations

The ethical endorsement was procured from King Faisal University's research ethics committee, bearing the ethical number KFU-REC-2023-MAY-ETHICS1148. Consent was sought and obtained, or in certain scenarios, waived from every participant. This ethical compliance accentuated voluntary involvement, data confidentiality, and sidestepping potential conflicts of interest.

Data analysis

SPSS software version 28.0 (IBM Corp., Armonk, NY) was the analytical tool of choice, enabling correlation and regression examinations and Chi-square testing to unearth connections between environmental interactions and pediatric asthma prevalence.

Study limitations

Inherent limitations comprised the dependence on participant self-reporting, potential recall biases, and the cross-sectional model's inability to conclusively attribute causation.

## Results

Demographic characteristics

Table 1 shows that a total of 1666 participants were included in the study, representing diverse demographic profiles. The majority of participants were within the 2-6 age range (52.5%), with females comprising 57.1%. Urban areas were the predominant residential location (84.5%).

**Table 1:** Demographic Characteristics Demographic Characteristics - Participant distribution by age, gender, city of residence, and geographic location

		Count	Column N %
Age	2-4	427	25.6%
	5-6	448	26.9%
	7-8	280	16.8%
	9-10	364	21.8%
	11-12	77	4.6%
	13-14	63	3.8%
Gender	Female	952	57.1%
	Male	707	42.4%
City of residence	Eastern Province	959	57.6%
	Middle Province	154	9.2%
	Northern Province	14	0.8%
	South Province	112	6.7%
	Western Province	420	25.2%
Geographic Location	Rural	63	3.8%
	Suburban	189	11.3%
	Urban	1407	84.5%

General questions regarding environmental exposures and pediatric asthma prevalence

Table 2 shows most experienced asthma symptoms rarely or never (70.6%). Air quality perceptions varied, with 41.6% rating it as "Good." Indoor smoking exposure was reported by 22.3%. Furthermore, 11.8% reported observing mold or dampness at home.

**Table 2:** General questions regarding environmental exposures and pediatric asthma prevalence. General Questions - Pediatric asthma prevalence, symptom frequency, air quality perceptions, indoor smoking, mold exposure, and cleaning habits.

		Count	Column N %
Have you or your child ever been diagnosed with a disease other than asthma?	No	1211	72.7%
	Yes	448	26.9%
How often does your child experience asthma symptoms (e.g., wheezing, coughing, chest tightness)?	Daily	56	3.4%
	A few times a week	126	7.6%
	Less than once a week	196	11.8%
	Once a week	105	6.3%
	Rarely or never	1176	70.6%
How would you rate the air quality in your residential area?	Excellent	147	8.8%
	Fair	455	27.3%
	Good	693	41.6%
	Poor	322	19.3%
	Very poor	42	2.5%
Do you or your family members smoke indoors?	No	1288	77.3%
	Yes	371	22.3%
How frequently is your child exposed to tobacco smoke at home?	Daily	147	8.8%
	A few times a week	91	5.5%
	Less than once a week	91	5.5%
	Never	1267	76.1%
	Once a week	63	3.8%
Are there any visible signs of mold or dampness in your home?	No	1463	87.8%
	Yes	196	11.8%
How often do you clean your living space (e.g., dusting, vacuuming)?	Daily	714	42.9%
	A few times a week	567	34.0%
	Less than once a week	105	6.3%
	Never	21	1.3%
	Once a week	252	15.1%
Are there any pets (e.g., cats, dogs) in your home?	No	1372	82.4%
	Yes	287	17.2%
How frequently does your child come into contact with animals (e.g., at friends' houses, parks)?	Daily	175	10.5%
	Few times a week	105	6.3%
	Less than once a week	161	9.7%
	Never	1134	68.1%
	Once a week	84	5.0%
How would you rate the cleanliness of your child's school environment?	Excellent	217	13.0%
	Fair	413	24.8%
	Good	707	42.4%
	Poor	273	16.4%
	Very poor	49	2.9%

Air pollution exposure

Table 3 shows participants indicated exposure to heavy traffic or busy roadways (56.7%) and identified industrial facilities nearby (28.2%). Ongoing construction activities near residential areas were reported by 59.2% of participants. Notably, 30.7% reported a correlation between asthma symptoms and specific times of the year.

**Table 3:** Air pollution exposure Air Pollution Exposure - Exposure to traffic, industrial facilities, construction, and seasonal asthma symptoms.

		Count	Column N %
Does your residential area have heavy traffic or busy roadways?	No	714	42.9%
	Yes	945	56.7%
Have you noticed any industrial facilities or factories near your residential area?	No	1190	71.4%
	Yes	469	28.2%
Are there any construction activities ongoing near your residential area?	No	672	40.3%
	Yes	987	59.2%
Does your child experience asthma symptoms more often during specific times of the year (e.g., during sandstorms, hot weather)?	No	1148	68.9%
	Yes	511	30.7%

Participants' mean Likert scores revealed that spending time outdoors in high air pollution areas (3.79) and using air purifiers (3.27) were common practices. Additionally, the perception of visible air pollution (3.65) and keeping windows open for fresh air (3.75) received moderate agreement (Table 4).

**Table 4:** Air pollution exposure (Likert analysis) Air Pollution (Likert) - Mean scores for outdoor exposure, air purifier use, visibility of pollution, window ventilation, and respiratory protection.

	N	Minimum	Maximum	Mean	SD
How frequently does your child spend time outdoors in areas with high air pollution levels (e.g., busy streets, construction sites)?	1666	1	5	3.79	.954
Do you use any air purifiers or filters at home?	1666	1	4	3.27	.832
Have you observed any visible signs of air pollution (e.g., smog, haze) in your residential area?	1666	1	5	3.65	1.012
How frequently do you keep your windows open for fresh air circulation?	1666	1	5	3.75	1.061
Does your child experience asthma symptoms more often during specific times of the year (e.g., during sandstorms, hot weather)?	1666	1	5	3.69	1.010
Do you use any respiratory protection (e.g., masks) for your child when air pollution levels are high?	1666	1	5	3.22	1.12

Multivariate (linear regression) model to identify the factors predicting the air pollution

Table 5 shows gender significantly influenced air pollution exposure, with females 1.54 times more likely to be exposed. Education level, employment status, and geographic location also exhibited significant associations (p < 0.001).

**Table 5:** Multivariate (linear regression) model to identify the factors predicting air pollution Multivariate (Air Pollution) - Odds ratios predicting air pollution exposure by gender, education, employment, and location.

Variable	Odds Ratio (OR)	95% Confidence Interval (CI)	P-value
Gender (Reference: Female)	1.54	1.20 - 1.98	<0.001
Education level			<0.001
- High school or less	1 (Reference)		
- Diploma	0.92	0.68 - 1.25	
- Bachelor’s degree	0.78	0.61 - 1.01	
- Master’s degree	0.60	0.42 - 0.85	
Employment Status			<0.001
- Employed full-time	1 (Reference)		
- Employed part-time	1.17	0.74 - 1.85	
- Retired	1.65	1.26 - 2.17	
- Student	1.32	1.06 - 1.64	
- Unemployed	1.14	0.88 - 1.47	
Geographic Location			<0.001
- Urban	1 (Reference)		
- Rural	1.32	0.85 - 2.04	
- Suburban	0.98	0.74 - 1.30	

Indoor allergen exposure

Allergen exposure factors were evaluated, with 50.8% reporting allergies to dust mites and 76.5% having carpets or rugs at home. Houseplants were present in 53.8% of homes, while pets were allowed in only 9.2% of children's bedrooms (Table 6).

**Table 6:** Indoor allergen exposure Indoor Allergen Exposure - Allergen-related factors, allergies, carpets, houseplants, pets, and cleaning practices.

		Count	Column N %
Is anyone in your household allergic to dust mites?	No	812	48.7%
	Yes	847	50.8%
Are there any carpets or rugs in your child's bedroom or living areas?	No	385	23.1%
	Yes	1274	76.5%
Do you have any houseplants in your home?	No	763	45.8%
	Yes	896	53.8%
Are there any pets allowed in your child's bedroom?	No	1505	90.3%
	Yes	154	9.2%
Do you use any dust mite covers for your child's mattress or pillows?	No	1155	69.3%
	Yes	504	30.3%
Does your child have any known allergies to specific allergens (e.g., pollen, pet dander)?	No	1302	78.2%
	Yes	357	21.4%

Table 7 shows Participants' mean Likert scores highlighted the frequency of cleaning practices, including bedding (3.59), carpet vacuuming (3.76), houseplants (3.70), soft furnishings (3.5), and sun exposure for bedding and soft furnishings (3.59).

**Table 7:** Indoor exposure of allergen (Likert analysis) Allergen (Likert) - Mean scores for bedding, carpet cleaning, houseplants, soft furnishings, and sun exposure practices.

	N	Minimum	Maximum	Mean	SD
How frequently do you clean your child's bedding (e.g., sheets, pillows) to prevent dust mite exposure?	1666	1	5	3.59	1.129
How often do you vacuum or sweep the carpets or rugs in your home?	1666	1	5	3.76	.998
Do you have any houseplants in your home?	1666	1	5	3.70	.944
How frequently do you clean your child's stuffed toys or soft furnishings?	1666	1	5	3.5	.988
How often do you air out your child's bedding and soft furnishings in the sun?	1666	1	5	3.59	1.129

Liner regression model between air pollution and allergen exposure

The predictor variable "Air pollution" demonstrated a significant positive relationship (B = 0.733, 95% CI = 0.612 to 0.970, p < 0.001) with allergen exposure (Table 8).

**Table 8:** Liner regression model between air pollution and allergen exposure

Predictor	Regression co-efficient (B)	95% CI of (B)	P-value
Air pollution	0.733	0.612, 0.970	<0.001

Multivariate (linear regression) model to identify the factors predicting the allergen exposure

Variables including Gender, Education level, Employment Status, and Geographic Location were analyzed. Gender (Odds Ratio (OR) = 1.12, 95% CI = 1.20 to 1.44, p < 0.001) and various education levels exhibited significant associations with allergen exposure (Table 9).

**Table 9:** Multivariate (linear regression) model to identify the factors predicting the allergen exposure Multivariate (Allergen) - Odds ratios predicting indoor allergen exposure by gender, education, employment, and location.

Variable	Odds Ratio (OR)	95% Confidence Interval (CI)	P-value
Gender (Reference: Female)	1.12	1.20 - 1.44	<0.001
Education level			<0.001
- High school or less	1 (Reference)		
- Diploma	0.88	0.68 - 1.16	
- Bachelor’s degree	0.78	0.61 - 1.01	
- Master’s degree	0.60	0.42 - 0.85	
Employment Status			<0.001
- Employed full-time	1 (Reference)		
- Employed part-time	1.113	0.74 - 1.85	
- Retired	1.69	1.26 - 2.17	
- Student	1.25	1.06 - 1.64	
- Unemployed	1.16	0.88 - 1.47	
Geographic Location			<0.001
- Urban	1 (Reference)		
- Rural	1.44	0.85 - 2.04	
- Suburban	0.55	0.74 - 1.30	

## Discussion

Our investigation sought to identify the prevalence of pediatric asthma in children within Saudi Arabia, leveraging an online and hard copy survey format. The perceived quality of air was rated as "Good" by 41.6% of participants. This aligns with a prior study in Rabigh City that reported similar percentages in asthma prevalence among its young demographic [11]. Notably, regional disparities in asthma prevalence were observed, with locations recording the highest rates, which have been attributed to various factors, including gender, diet, household pets, and environmental interactions.

Central to our inquiry was understanding the link between potential environmental risk factors and pediatric asthma. Noteworthy findings include a 56.7% exposure to traffic-congested zones and 28.2% proximity to industrial settings. Additionally, 59.2% reported ongoing construction activities in their vicinities, and a significant 30.7% observed seasonality in asthma symptoms. This observation is bolstered by a Cleveland study which found increased pediatric asthma-related doctor visits in areas with elevated air pollution [12]. Furthermore, research from Riyadh solidified the connection between heightened serum polycyclic aromatic hydrocarbon (PAH) levels and childhood asthma [13]. Factors like respirable particulate matter, black carbon, nitrogen dioxide, and ozone were associated strongly with pediatric asthma, particularly in those under regular corticosteroid treatment [13-15]. Intriguingly, our data suggests a gender bias, with females 1.54 times more likely to experience heightened air pollution exposure. Socio-economic variables such as educational attainment, occupation, and residence also had significant ties to exposure levels (p < 0.001).

Delving into the well-documented correlation between allergies and asthma [11,13], our research found that 50.8% were allergic to dust mites and 76.5% had carpets at home. Interestingly, only 9.2% allowed pets in the children's rooms. Reinforcing our observations, a study from Southern Florida found a notable percentage of children diagnosed with asthma also had heightened sensitivity to common allergens [16]. Another Kuwait-based study emphasized the connection between pet ownership and allergic manifestations, including asthma [17]. In our context, sensitization to dust mites was more indicative of childhood asthma than pet ownership. Key demographic variables such as gender and educational levels also had substantial associations with allergen exposure.

However, it's paramount to note this study's limitations. Potential recall bias is inherent in self-administered questionnaires. While we utilized a reliable tool, there remains the possibility of inaccuracy in some responses. The participant demographic, limited to parents engaging in the online survey, may not offer a holistic representation. Furthermore, the cross-sectional nature of the study design hinders our ability to derive cause-and-effect relationships between environmental and allergen exposure vis-à-vis asthma occurrence.

## Conclusions

The present study embarked on a detailed exploration of pediatric asthma within Saudi Arabia, emphasizing the roles of environmental influences and allergen exposures. Our findings resonate with existing literature, pinpointing regional variances in asthma prevalence. Furthermore, our data underscores strong links between environmental determinants, notably heavy traffic and industrial vicinity, with pediatric asthma manifestation. Household allergens, especially dust mites and specific carpet types, emerged as substantial contributors to the disease's prevalence. In addition, socio-economic and gender dynamics demonstrated noteworthy impacts on the susceptibility and severity of asthma symptoms in children. While our methodology predominantly leaned on self-reported questionnaires, introducing a potential bias, and its cross-sectional framework curtailed in-depth causative insights, it laid the groundwork for refined future investigations. To truly augment the literature and provide transformative insights, there's an imperative for more granular, longitudinal studies, potentially utilizing advanced techniques and larger, diversified samples. Such future endeavors can pave the way for crafting tailored public health interventions, addressing the specific needs of the pediatric population in Saudi Arabia concerning asthma.
